# Evaluation of Circulating and Archived HIV-1 Integrase Drug-Resistance Variants among Patients on Third-Line ART in Cameroon: Implications for Dolutegravir-Containing Regimens in Resource-Limited Settings

**DOI:** 10.1128/spectrum.03420-22

**Published:** 2022-10-19

**Authors:** Joseph Fokam, Ezechiel Ngoufack Jagni Semengue, Evariste Molimbou, Naomi-Karell Etame, Maria Mercedes Santoro, Désiré Takou, Leonella Mossiang, Alain Patrice Meledie, Collins Ambe Chenwi, Bouba Yagai, Alex Durand Nka, Beatrice Dambaya, Georges Teto, Aude Christelle Ka’e, Grâce Angong Beloumou, Sandrine Claire Djupsa Ndjeyep, Nadine Fainguem, Aissatou Abba, Aurelie Minelle Ngueko Kengni, Michel Carlos Tommo Tchouaket, Nounouce Pamen Bouba, Serge-Clotaire Billong, Rina Djubgang, Edith Temgoua Saounde, Samuel Martin Sosso, Charles Kouanfack, Anne-Cecile Zoung-Kanyi Bissek, Emmanuel Eben-Moussi, Vittorio Colizzi, Carlo-Federico Perno, Francesca Ceccherini-Silberstein, Alexis Ndjolo

**Affiliations:** a Chantal BIYA International Reference Centre for Research on HIV/AIDS Management and Care, Yaoundé, Cameroun; b Faculty of Health Sciences, University of Buea, Buea, Cameroon; c Faculty of Medicine and Biomedical Sciences, University of Yaoundé I, Yaoundé, Cameroun; d National HIV Drug Resistance Working Group, Ministry of Public Health, Yaoundé, Cameroun; e University of Rome Tor Vergata, Rome, Italy; f Evangelical University of Cameroon, Bandjoun, Cameroon; g School of Health Sciences, Catholic University of Central Africa, Yaoundé, Cameroun; h Yaoundé Central Hospital, Yaoundé, Cameroun; i Douala General Hospital, Douala, Cameroon; j Mvangan District Hospital, Mvangan, Cameroon; k PhD Courses in Microbiology, Immunology, Infectious Diseases and Transplants (MIMIT), University of Rome “Tor Vergata”, Rome, Italy; l Directorate for Disease, Epidemic and Pandemic Control, Yaounde, Cameroon; m Central Technical Group, National AIDS Control Committee, Yaoundé, Cameroun; n Directorate of Pharmacy, Drug and Laboratory, Ministry of Public Health, Yaoundé, Cameroun; o Faculty of Medicine and Pharmaceutical Sciences, University de Dschang, Dschang, Cameroon; p Division of Operational Health Research, Ministry of Public Health, Yaoundé, Cameroun; q Bambino Gesu Pediatric Hospital, Rome, Italy; Fundacio irsiCaixa

**Keywords:** archived resistance, third-line ART, dolutegravir, raltegravir, Cameroon, HIV-1, integrase inhibitors

## Abstract

To ensure the long-term efficacy of dolutegravir (DTG), we evaluated the genotypic profile in viral reservoirs among patients on third-line (3L) antiretroviral therapy (ART) in Cameroon, according to prior exposure to raltegravir (RAL). A facility-based study was conducted from May through December 2021 among patients on 3L ART from HIV treatment centers in Yaoundé and Douala. Viral load was measured, and genotyping was performed on plasma RNA and proviral DNA. HIV-1 drug resistance mutations were interpreted using HIVdb.v9.1 and phylogeny analysis was performed using MEGA.v7, with *P* < 0.05 considered significant. Of the 12,093 patients on ART, 53 fully met our inclusion criteria. The median (IQR) age was 51 years (40 to 55 years), and the male/female ratio was 4/5. The median duration on integrase strand-transfer inhibitors (INSTI)-containing regimens was 18 months (12 to 32 months), and 15.09% (8/53) were exposed to RAL. The most administered 3L ART was TDF+3TC+DTG+DRV/r (33.96%, 18/53). Only 5.66% (3/53) had unsuppressed viremia (>1000 copies/mL). Resistance testing in proviral DNA was successful for 18/22 participants and revealed 1/18 patients (5.56%, in the RAL-arm) with archived mutations at major resistance positions (G140R and G163R). Five subtypes were identified, CRF02_AG (12/18), CRF22_01AE (3/18), A1 (1/18), G (1/18), and F2 (1/18). In Cameroon, 3L-experienced patients had a good virological response with a low level of archived mutations in the integrase. This finding underscored the use of DTG-containing ART for heavily treated patients in similar programmatic settings. However, patients with prior exposure to RAL should be closely monitored following a stratified or personalized approach to mitigate risks of INSTI-resistance, alongside pharmacovigilance.

**IMPORTANCE** We described the analysis of the genotypes of the population within third-line antiviral therapy in Cameroon, with a focus on defining the effects of prior raltegravir (RAL) treatment and resistance mutations for current dolutegravir (DTG) treatment. While supporting the current transition to DTG-containing ART in resource-limited settings toward the achievement of the UNAIDS’ goal of HIV elimination by 2030, our findings suggested that RAL-exposed patients may need a specific monitoring approach either in a stratified or personalized model of third-line ART to ensure the long-term success of DTG-containing regimens.

## INTRODUCTION

Infection with the human immunodeficiency virus (HIV) requires a lifelong antiretroviral therapy (ART) (1–5), and the roll-out of ART has significantly reduced HIV-related mortality and improved the quality of life of people living with HIV (PLHIV) (1, 6–9). Before 2020, the ART strategy in several resource-limited settings (RLS), including Cameroon, was based on a public health approach that recommended the use of nonnucleoside reverse transcriptase inhibitors (NNRTIs) in combination with two NRTIs as a first-line regimen, ritonavir-boosted protease inhibitors (PI/r) in combination with two NRTIs as second-line, and ritonavir-boosted darunavir (DRV/r, a second-generation PI/r) and/or integrase strand transfer inhibitors (INSTI) as third-line ART guided by genotyping (10–14). However, despite progress recorded in ART programs in treatment coverage and earlier initiation of therapy among people living with HIV (PLHIV), achieving the goals of ending AIDS as an epidemic remains challenging in RLS, especially with barriers to health care in the frame of the COVID-19 pandemic (2, 3,15–17). In this context, poor retention in care, nonadherence or treatment interruption, and events of discontinuity in drug supply, favor viral rebound and risks of HIV drug resistance (HIVDR) in RLS (15, 18–24). This is even more concerning in settings where NNRTI-based regimens remain the preferred prescribed ART due to their low genetic barrier to resistance. Of note, HIVDR gives mutant viruses a selective advantage over wild-type HIV strains, which enables them to replicate even in the presence of antiretrovirals (ARVs) (25), leading to poor virological outcomes and increased mortality and morbidity (26).

With the increasing burden of pretreatment drug resistance above 10% (driven by NNRTI resistance) in several RLS, countries were encouraged to either initiate NNRTI-based ART guided by genotypic resistance testing (wherever applicable) or transition from NNRTI- to dolutegravir (DTG)-based first-line ART (27–35). Moreover, for patients failing second-line ART, DTG remains highly recommended as part of a third-line combination in RLS (27–35). DTG is a second-generation INSTI that has a greater efficacy over other INSTIs, due to its high potency (i.e., viral inhibitory capacity), its limited risk of cross-resistance with raltegravir (RAL) and elvitegravir (EVG), its tolerability, its high genetic barrier to resistance (i.e., requires several mutations to achieve a loss of activity), and its high forgiveness of nonadherence (1,34, 36). Nonetheless, in the frame of previous exposure to first-generation INSTI, we hypothesized that persistent viral replication under RAL (or even EVG) may prompt the selection of INSTI-DRMs, which could jeopardize treatment efficacy in a patient receiving subsequent therapies containing DTG (37–39). If proven, the predicted benefits from current WHO’s recommendations, applied to patients with prior exposure to RAL or EVG, could be threatened by the presence of acquired INSTI-DRMs archived in viral reservoirs. In a context where management of treatment-experienced HIV-infected patients remains challenging, drug options and access to genotypic resistance testing (GRT) are limited. Poor adherence and lost-to-follow-up are frequent, and programmatic monitoring remains suboptimal (39–42). Developing a public health model guided by the history of RAL/EVG exposure may lead to a stratified use of DTG to ensure maximal and long-term efficacy of third-line ART in RLS. Our study objective was to characterize the patterns of INSTI-DRMs in viral reservoirs of patients on third-line DTG-containing regimens according to previous exposure to RAL in Cameroon.

## RESULTS

### Sociodemographic characteristics.

Out of 12,093 patients receiving ART at the reference treatment centers (Yaoundé Central Hospital and Douala General Hospital), 53 (0.44%) patients fully met our inclusion criteria of being on a third-line ART regimen. The study population was composed of 54.71% (29/53) women. The median age was 51 years (interquartile range [IQR], 40 to 55 years), and 58.49% (31/53) of the study population resided in the central region of Cameroon.

### Clinical characteristics.

The median (IQR) viremia before initiation of third-line ART was 3,795 copies/mL (220 to 169,322 copies/mL) while the median CD4 count was 157 cells/mm^3^ (84 to 285 cells/mm^3^). The overall median duration of ART since treatment initiation was 192 months (162 to 222 months). Specifically, for third-line ART, the most administered regimen was TDF (tenofovir)+3TC (lamivudine)+DTG +DRV/r (ritonavir boosted darunavir) (33.96%; 18/53), followed by TDF+3TC+DTG (22.64%; 12/53). The median treatment duration under the INSTI-containing regimen was 18 months (12 to 32 months), with 15.09% (8/53) of the participants having a documented exposure to RAL. Table 1 summarizes the baseline features of study participants.

**TABLE 1:** Baseline characteristics of study participants

Characteristic	Overall	Participants with prior exposure to RAL	Participants unexposed to RAL
Total, *n* (%)	53 (100.0)	8 (15.1)	45 (84.9)
Gender distribution, *n* (%)			
Female	29 (54.72)	3 (37.5)	26 (57.78)
Male	24 (45.28)	5 (62.5)	19 (42.22)
Median age (interquartile range), yrs	51 (40–55)	51 (48–53)	48 (40–58)
Residence, *n* (%)			
Yaoundé	31 (58.49)	6 (75)	25 (55.56)
Douala	22 (41.51)	2 (25)	20 (44.44)
Median viremia before third-line initiation (interquartile range),HIV-1 RNA copies/mL	3795 (220–169,322)	13929 (2,724–15,916)	2724 (199–101,030)
Median CD4 count before third-line initiation (interquartile range),cells/mm^3^	157 (84–285)	195 (149 – 804)	148 (75 – 226)
Median ART-duration (interquartile range), mo	192 (162 – 222)	192 (162 – 202)	184 (162 – 222)

### Virological response after exposure to INSTI.

Among these patients on third-line ART, the overall rate of viral suppression (<1000 copies/mL) was 94.34% (50/53). Interestingly, the median (IQR) viremia was <40 copies/mL (00 to <40 copies/mL), indicating an optimal viral control in the study population. Specifically, 75.47% (40/53) of the participants had a controlled viremia (<40 copies/mL), and 24.53% (13/53) had a detectable viremia, further stratified into 18.86% (10/53) with detectable but suppressed viremia (40 to 999 copies/mL) and 5.66% (3/53) with unsuppressed viremia (2,392 [3.4 log], 17,461 [4.2 log], and 144,148 [5.2 log] copies/mL, respectively).

### Genotypic resistance testing.

**(i) INSTI resistance in plasma RNA.** Of the 13 patients on INSTI with detectable viral load (i.e., considered replicative infection), plasma RNA amplification and sequencing were effective on the three (03) with viremia ≥1000 copies/mL as per standard of care. These samples were from patients exposed to DTG at the moment of the study but without any history of exposure to RAL. Among these patients, no major mutation affecting INSTIs in plasma RNA was found, except an accessory mutation (L74I) detected in 2/3 (66.67%).

**(ii) INSTI resistance in proviral DNA.** Of the 53 patients included in our study, amplification and sequencing of proviral DNA were effective in 18/22 (81.81%), of whom 44.44% (8/18) had documented exposure to RAL. Among unsuccessful amplified samples, three had plasma viremia <40 copies/mL and one had a plasma viremia of 76 copies/mL. Archived mutations were observed in 1/18 (5.56%) patients (in the RAL-exposed arm) at major drug resistance positions for RAL and EVG (G140R and G163R). Other accessory mutations (L74I [27.78%; 5/18] and E157Q [5.56%; 1/18]) were found only in patients unexposed to RAL, as natural polymorphisms in the integrase coding region (see Table 2).

**TABLE 2:** Summary of archived mutations and polymorphisms found in viral reservoirs

Patient	Exposure to RAL	Major DRMs	Accessory DRMs	Polymorphisms
1	No	None	E157EQ	K14R, W19W*, V31I, L45LI, K46KQ, L101I, T112V, S119P, T122TI, T124A, T125A, W132W*, G134N, K136T, D167E, S195T, V201I, T206S, L234I, R269RK, S283G, D286DN
2	Yes	G140R, G163R		G4R, E11D, K14R, A21T, A23V, D41N, G47R, M50I, I72V, L101I, T112V, S119T, T124N, T125A, G134N, K136Q, R166K, D167E, V201I, R224Q, L234V, S255D, G272R, S283G, D288N
3	No	None	L74I	S17N, R20RK, V31I, L63IV, I72V, K111R, T112V, T124A, T125A, G134N, K136T, D167E, T174TP, V201I, T206S, I220IV, L234I, R269K, R284G, D286N
4	No	None	L74I	L2LF, K14R, V31I, I72V, L101I, T112V, T124A, T125A, G134N, I135V, K136T, V201I, T206S, R224RW, R228RK, P233PS, L234I, R269K
5	Yes	None		K14R, S24N, V31I, L101I, G134N, K136T, K173R, T174TP, V201I, T206S, T218S, L234I, S255N, D256E, S283G
6	No	None		K14R, S17N, L28I, V31I, S39C, I72V, T112V, T124A, T125A, K136T, S153SA, L158I, T174TP, G197GV, V201I, T206S, T218I, L234I, S283SG, D288DE
7	Yes	None		E11D, K14R, S17T, R20K, V31I, L101I, T112V, T124A, T125A, G134N, I135V, K136T, V201I, T206S, Q221QK, R224RW, Y227F, P233PS, L234I, L241LV, L242LV, W243WR, S283G
8	No	None	L74I	E11D, K14R, A21T, V31I, I72V, A91G, Y99F, L101I, T112V, T124A, T125A, G134N, I135V, K136T, V201I, T218S, R224RW, P233PS, L234I, W235WR, L241LV, L242LV, W243WR, V250VI, D256DE, D270DV
9	No	None		K14R, A23AV, V31I, S57SG, L101I, T112IV, T124A, T125A, G134N, I135V, K136T, V201I, K215KT, R224RW, P233PS, L234I, L241LV, D256DE, I268IF, S283G, R284G, D288N
10	Yes	None		E11D, K14R, S17C, A23V, V31I, I72V, L101I, T112V, T124A, T125A, G134N, I135V, K136T, V201I, T206S, T218TS, R224RW, P233PS, L234I, S255SR, I268IF, D278DE, V281VG, S283SG, D288DE
11	No	None	L74I	L2F, S17N, V31I, L101I, T112V, T124A, T125A, G134N, I135V, K136T, V201I, T206S, L234I, D256E, R269K, D278N, S283G, R284RG
12	No	None	L74I	D6E, K14R, S17N, D25E, V31I, I60V, L63I, I72V, T97S, L101I, T112V, S119T, T124A, T125A, G134N, I135V, K136T, K188R, V201I, I203M, T218S, L234I, R269K, K273Q, D278A, S283G, D288N
13	Yes	None		K14R, S17SN, V31VI, I72IV, L101LI, L104LV, T112I, T124A, T125AV, G134N, I135V, K136T, V201I, T206TS, L234I, D256DE, R269RK, S283G
14	Yes	None		K14R, S17SN, S24SN, S39SN, M50MI, L101I, L104V, T112V, I113IV, S119ST, T124DN, T125A, I135IV, K136T, V201I, T206TS, R224RW, L234I, D256DE, K264KE, S283G
15	Yes	None		E11D, K14R, A21T, A23V, V31I, M50I, T93N, L101I, T112V, T124A, T125A, G134D, K136Q, D167E, V201I, T218S, L234I, S283G, D288N
16	Yes	None		E10EQ, E11D, K14R, V32I, D41N, I72V, L101I, T112V, T124A, T125A, G134D, K136Q, D167E, V201I, L234I, S283G, R284G, D286E, D288N
17	No	None		K14R, K34R, I72IV, E85EK, L101I, T112V, I113IV, T124A, T125A, A133AT, G134N, I135V, K136T, V201I, T206S, L234I, M275V, S283G
18	No	None		S17N, L45P, K103R, T112V, I113V, S119P, T124N, T125V, K136Q, V201I, T206TS, L234I, D256E, S283G

### Genetic diversity.

**FIG 1:**
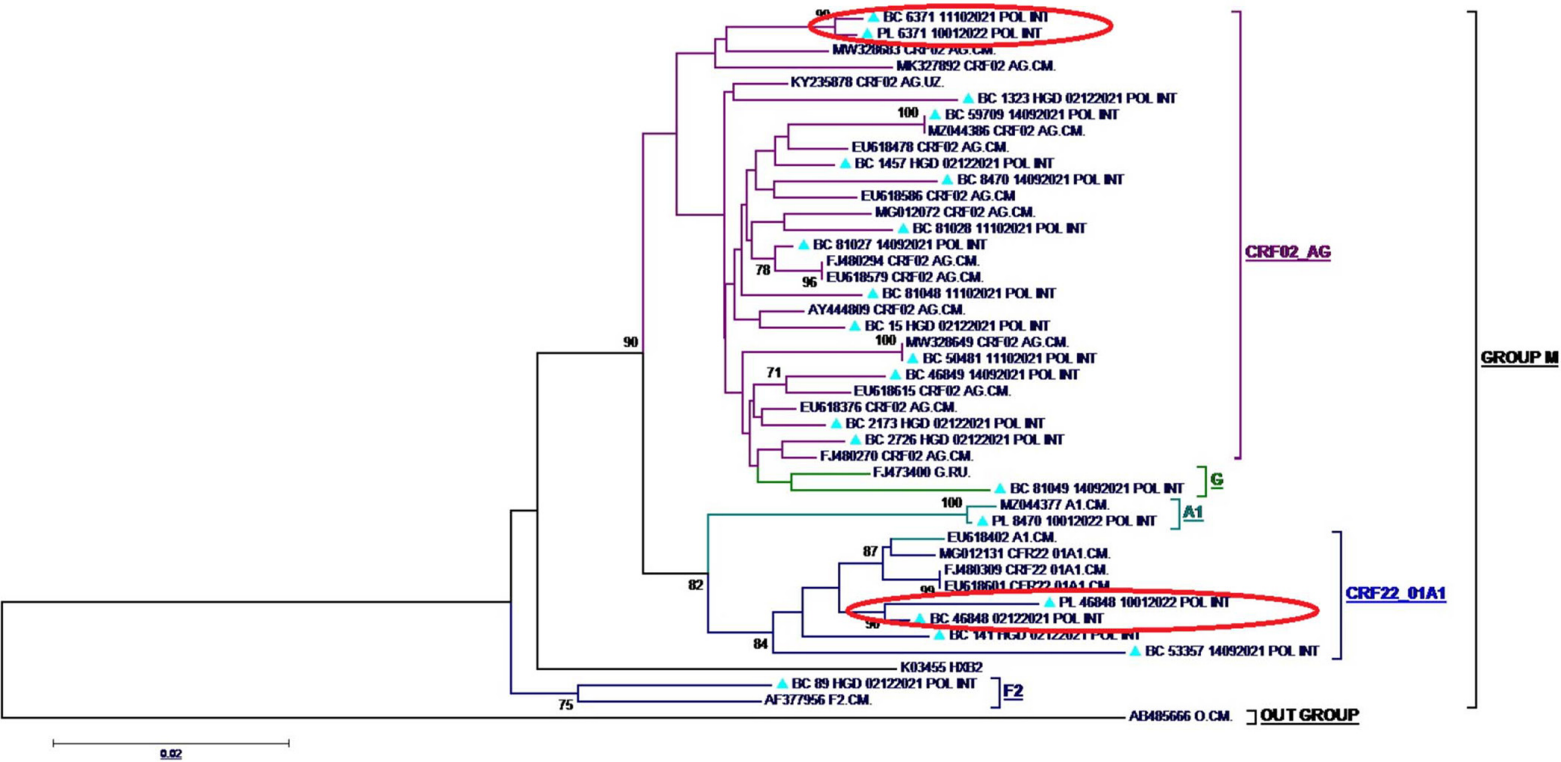
Phylogenetic tree of sequences generated. Triangles, patients' derived sequences; PL, the sequence obtained from a plasma RNA sample; BC, the sequence obtained from proviral DNA; ovals, sequences of RNA and DNA from the same patients.

In the plasma RNA of the study population, sequences obtained revealed the presence of the recombinant forms CRF02_AG (1/3), CRF22_01AE (1/3), and the pure subtype A1 (1/3). In proviral-DNA, five HIV-1 subtypes were detected: the recombinant form CRF02_AG (66.67%; 12/18), followed by CRF22_01AE (16.67%; 3/18), and the pure subtypes A1 (5.56%; 1/18), G (5.56%; 1/18) and F2 (5.56%; 1/18), as shown in Fig. 1.

## DISCUSSION

The current transition to a DTG-based ART is predicted to be very effective in rapidly controlling viral replication, which would limit the transmission of resistant viruses and, thus, lead to a control of the epidemic (27–35). To prevent DTG resistance in third-line ART, we analyzed the genotypic profile in proviral DNA among 53 patients in the Cameroonian routine clinical setting. The median age was 51 years with similar sex distribution and about 16 years of ART experience. To better delineate the clinical relevance of INSTI, we analyzed the potential effect of prior exposure to RAL on the risk of acquiring DRMs to DTG.

The described demographics feat with the local HIV epidemics, thus supporting the representativeness of our findings to the target population (14, 43–46). In effect, from a total of more than 12.000 patients monitored in our routine clinical practice, we carefully screened for participants fully complying with all study criteria. With less than 1% of patients on third-line regimens, our findings are comparable with current knowledge and thus serve as advocacy for enhanced detection of patients in need of third-line ART regimens (14, 44–47). Furthermore, about 15% of patients on third-line were previously exposed to RAL, indicating a potential risk of cross-resistance to DTG that supports the current investigation for optimal care in the clinical setting.

Regarding baseline immuno-virological profiles (before third-line initiation), most patients had a moderate viral replication (around 3 log copies/mL) and poor immunity (CD4 < 200 cells/mm^3^), suggesting an overall poor clinical condition for these patients, and reduced viral replicative fitness (25, 48), before initiation of DTG-based third-line ART. Of relevance, once switched to third-line, these DTG-containing ART regimens were very effective, translated by 94% viral suppression (<1,000 copies/mL) and 75% virological control (<40 copies/mL) after approximately 18 months on third-line. This result indicates a very good and rapid response to the used third-line ART, which correlates with recent findings (33, 49–52), and strongly supports the current use of the WHO-recommended third-line ART strategy in RLS (27–35). Of note, the most prescribed third-line ART was a four-drug combination (TDF+3TC+DTG+DRV/r), in line with common prescribing practices for third-line regimens in several countries (35, 39, 45, 53–55).

Regarding the genotypic profile, few samples among RAL-unexposed participants were randomly selected to feat with the low proportion of RAL-exposed to compare the results in both arms. We had an overall high-performance rate of amplification and sequencing in the proviral DNA. Resistance testing in proviral DNA revealed the presence of G140R and G163R archived in viral reservoirs of a patient in the RAL-arm, as well as the presence of accessory mutations (L74I, T97A, and E157Q) among RAL-unexposed third-line patients. It is worth noting that G140R – when found in the plasma – is associated with an intermediate-level of resistance to cabotegravir (CAB) and is a revertant mutation from the original G140S/A/C, which are nonpolymorphic mutations that usually occur with Q148 mutations. Alone, G140S/A/C has minimal effects on INSTI susceptibility. However, in combination with Q148 mutations, they are associated with high-level resistance to RAL and EVG and intermediate reductions in DTG and BIC susceptibilities. The appearance of G140R henceforth suggests that Q148 mutation(s) may have been previously selected at the population level under RAL pressure (34, 56, 57). G163R is an accessory polymorphic mutation in subtype F viruses from ARV-naive patients, which is rather nonpolymorphic in other viral clades. Alone, G163R has little effect on RAL and EVG susceptibility but does not affect CAB, DTG, and BIC (34, 56, 57). Despite all these facts, G140R and G163R are hard to relate to resistance in the present case given that they may have resulted as a consequence of the apolipoprotein B mRNA-editing enzyme, catalytic polypeptide-like 3 (APOBEC3) mutagenesis activity in the proviral DNA (58, 59). In effect, APOBEC3 has been studied intensely in the field of virology because it was recognized early on to deaminate cytosines in cDNA reverse transcription intermediates of retroviruses, including HIV-1 (58–60), thus leading to mutations not always related to drug-pressure. Furthermore, even though the impact of these ambiguous mutations on DTG-based ART is not concerning, the potential presence of other major DRMs in minority viral populations (<20% sequencing threshold) needs to be ascertained (61, 62). In a context where drug options are exhausted after a third-line ART, the current evidence suggests either personalized therapeutic management (i.e., ART combination guided by genotyping) or a stratified management approach of patients on third-line to prevent the emergence of INSTI-resistance, as we earlier reported in the same country (39). This measure may also pave the way to limit the risk of a new epidemic of multidrug-resistant viruses in RLS (39, 54, 55, 63). Nonetheless, ensuring the success of the programmatic strategy in RLS would require further investigations to implement the best (cost-effective) approach, especially considering all the challenges of resource-limited countries (14, 21).

Finally, the most represented viral clade in this study was the recombinant form CRF02_AG, similar to several reports in Cameroon (64–69). Of note, the broad HIV diversity found in this small sample population could justify the considerable number of polymorphisms observed in viral reservoirs (25, 66). Long-term monitoring might help in depicting the potential effects of covariation of these mutants on treatment outcomes under DTG-containing regimens. The fewer cases of patients exposed to RAL (about 15%) reflects the real-life situation of RAL use and suggests the need for long-term surveillance in this group of patients, despite the possibility of a reduced proportion of patients with RAL exposure over time in the era of DTG scale-up in third-line and on-going phase-out of RAL and EVG from national guidelines due to their low genetic barrier to resistance. In perspective, combining pharmacovigilance studies would be essential in appraising the effectiveness toward a long-term definition of the benefits and risks of DTG-containing ART in diverse populations (adults, adolescents, children) and drug formulations in RLS (17, 35, 70).

In conclusion, our evidence suggested a high efficacy of DTG-containing third-line ART in Cameroon, supporting the current WHO’s recommendations and the achievement of the UNAIDS’ goal of HIV elimination by 2030. The low level of archived mutations at integrase-resistance positions in a patient on dolutegravir, previously exposed to raltegravir, underscores the need for a specific monitoring approach for such patients, either in stratified or personalized monitoring model of third-line ART. In perspective, pharmacovigilance components would be complementary for assessing the long-term cost-effectiveness of this highly potent regimen.

## MATERIALS AND METHODS

### Study design and patients.

We conducted a prospective and facility-based study from May through December 2021 among HIV-infected patients followed up at the Douala General Hospital (HGD) and the Yaoundé Central Hospital (HCY). These accredited treatment centers were chosen because they have the largest cohorts of PLHIV in Cameroon. Any patient currently on third-line ART with documented exposure to INSTIs was considered for inclusion. Demographic and clinical information such as sex, age, ART, and duration of treatment was collected from patient records. Laboratory analyses within the frame of the study were performed at the Chantal Biya International Reference Centre for research on HIV/AIDS prevention and management (CIRCB) in Yaoundé, Cameroon.

The CIRCB is a government institution of the Ministry of Public Health dedicated to HIV research and patient monitoring in several aspects, among which HIV early infant diagnosis in the frame of the national PMTCT program; diagnosis of coinfections with HIV; viral load measurement; CD4 and CD8 T lymphocytes counts; biochemical and hematological tests for drug safety; genotypic HIVDR testing (GRT) at subsidized costs with quality control programs conducted in partnership with Quality Assessment and Standardization of Indicators (QASI) and other international agencies (http://www.circb.cm/btc_circb/web/).

### Measurement of viral load.

HIV-1 RNA quantification was performed on plasma samples using Abbott *m2000rt* real-time HIV platform (Abbott Molecular Inc., IL, USA) according to the manufacturer's instructions (https://www.molecular.abbott/int/en/products/infectious-disease/realtime-hiv-1-viral-load). Briefly, a protocol using 0.6 mL of plasma was used for RNA extraction, followed by simultaneous amplification and detection on a real-time PCR (RT-PCR). The lower and upper detection limits of the test were <40 and >10,000,000 copies of HIV-1 RNA copies/mL, respectively.

### HIV-1 genotypic drug resistance testing.

HIV-1 genotypic resistance testing (GRT) was performed on plasma (HIV-1 RNA) and Buffy coat (proviral DNA) aliquots using a highly sensitive integrase genotyping assay (65). Briefly, viral nucleic acids were extracted using the Qiagen protocol with a viral RNA extraction kit for plasma and viral DNA extraction for buffy-coat specimens. For RNA extracts, reverse transcriptase and PCR were performed using the following conditions (1 cycle at 50° for 30 min, 1 cycle at 94°C for 2 min; 40 cycles [95°C, 30 s; 51°C, 30 s; 72°C, 2 min, and 30 s]; 1 cycle at 72°C for 10 min; 1 cycle at 4°C for 30 min and 1 cycle at 10°C forever); for DNA extracts, a direct PCR was performed using the following conditions (1 cycle at 94°C for 12 min; 40 cycles [95°C, 30 s; 51°C, 30 s; 72°C, 2 min, and 30 s]; 1 cycle at 72°C for 10 min; 1 cycle at 4°C forever). Gel electrophoresis, purification, and sequencing reaction were performed similarly.

### Data processing and statistical analysis.

All generated sequences (plasma RNA and proviral DNA) were aligned in BioEdit version 7.2.6 (Tom Hall, Raleigh, NC) and compared to reference sequences available in the Los Alamos database (http://www.hiv.lanl.gov); gaps were then removed from the final alignment. The phylogenetic tree was inferred using the maximum likelihood method on MEGA v7.0.26 software for subtyping and to ensure that there was no cross-contamination of samples. The statistical robustness and reliability of the branching order in the phylogenetic tree were confirmed by a bootstrap analysis using 1,000 replicates on a maximum likelihood tree obtained by molecular phylogeny. Regarding the analysis of drug resistance mutations (DRMs), sequences obtained after capillary electrophoresis on the 3500 Genetic Analyzer (Applied Biosystems, USA) were assembled and manually edited using RECall (CDC, Atlanta, GA, USA). DRMs were interpreted using the Stanford HIVdb version 9.0 algorithm (https://hivdb.stanford.edu/hivdb/by-sequences/) and all mutations associated with INSTI resistance were listed. Data were entered, coded, and analyzed using Epi info version 7. The parameters of central tendency (mean and median) and dispersion (standard deviation and interquartile range) were used to describe the continuous variables. The Chi-square test (or Fisher test wherever appropriate) was used to compare frequencies, with a significance level of *P* < 0.05.

### Data availability.

Integrase sequences generated from proviral DNA in this study are available in GenBank under the accession numbers OP508478 to OP508495.
